# Effective Visible Light Exploitation by Copper Molybdo-tungstate Photoanodes

**DOI:** 10.1021/acsaem.0c01021

**Published:** 2020-06-08

**Authors:** Annalisa Polo, Chiara Nomellini, Ivan Grigioni, Maria Vittoria Dozzi, Elena Selli

**Affiliations:** Dipartimento di Chimica, Università degli Studi di Milano, via Golgi 19, I-20133 Milano, Italy

**Keywords:** Mo-modified CuWO_4_, band gap reduction, extended visible light photoactivity, photoelectrocatalysis, photoanode, charge separation

## Abstract

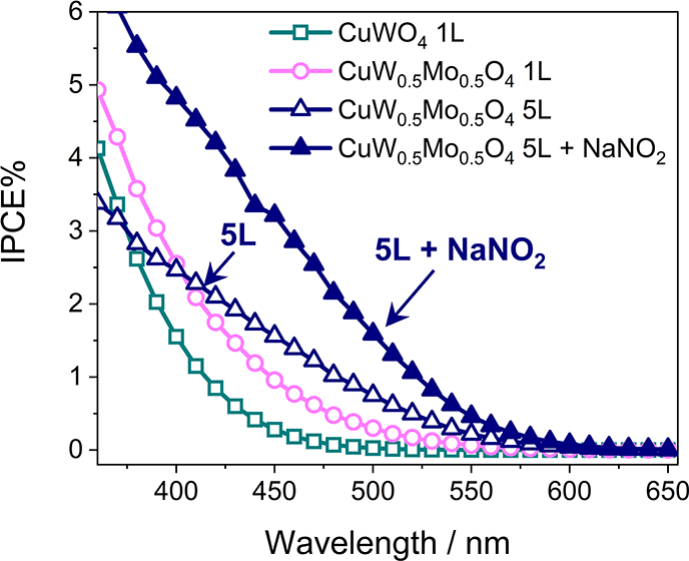

The need for stable oxide-based semiconductors with a narrow band gap, able to maximize the exploitation of the visible light portion of the solar spectrum, is a challenging issue for photoelectrocatalytic (PEC) applications. In the present work, CuW_1–*x*_Mo_*x*_O_4_ (*E*_g_ = 2.0 eV for *x* = 0.5), which exhibits a significantly reduced optical band gap *E*_g_ compared with isostructural CuWO_4_ (*E*_g_ = 2.3 eV), was investigated as a photoactive material for the preparation of photoanodes. CuW_0.5_Mo_0.5_O_4_ electrodes with different thicknesses (80–530 nm), prepared by a simple solution-based process in the form of multilayer films, effectively exhibit visible light photoactivity up to 650 nm (i.e., extended compared with CuWO_4_ photoanodes prepared by the same way). Furthermore, the systematic investigation on the effects on photoactivity of the CuW_0.5_Mo_0.5_O_4_ layer thickness evidenced that long-wavelength photons can better be exploited by thicker electrodes. PEC measurements in the presence of NaNO_2_, acting as a suitable hole scavenger ensuring enhanced photocurrent generation compared with that of water oxidation while minimizing dark currents, allowed us to elucidate the role that molybdenum incorporation plays on the charge separation efficiency in the bulk and on the charge injection efficiency at the photoanode surface. The adopted Mo for W substitution increases the visible light photoactivity of copper tungstate toward improved exploitation and storage of visible light into chemical energy via photoelectrocatalysis.

## 1 Introduction

Our need of storable forms of energy is continuously growing,^1,2^ as well as the attention toward renewable energy sources, in order to prevent crucial fossil-fuel-related issues such as climate change and air pollution. The sun is the primary source of energy for our planet, and the evolution toward a solar energy society, already envisaged long time ago, has now become an inescapable choice.^3^ Mimicking the action of bacteria and plants which are able to convert solar energy into nutrients via photosynthesis,^4^ effective strategies were foreseen to convert solar light into energy-containing chemicals.^1,4−6^ Among them, the photoelectrocatalytic (PEC) conversion of solar light into highly energetic chemical fuels was proposed, in thermodynamically uphill reactions such as hydrogen production from water splitting,^7,8^ and intense efforts were made in identifying photoactive materials for the fabrication of efficient photoelectrodes, in particular photoanodes for water oxidation, as this reaction represents the kinetic bottleneck of the overall PEC water splitting process.^9^

Ternary oxides, such as BiVO_4_, MFe_2_O_4_ (M = Cu, Mg, Zn), InTaO_4_, and CuWO_4_, have emerged as promising candidate materials for this application^10−17^ because of their high stability toward the harsh oxidative conditions required for oxygen evolution,^18^ their valence band edge position being lower in energy with respect to the water oxidation potential of 1.23 V versus RHE.^19^ Commonly employed BiVO_4_ and MFe_2_O_4_ oxides with a relatively narrow band gap may allow up to 8 and 10% harvesting of the solar radiation, respectively, which is still not suitable for industrial applications.^13,19^ However, their efficient use as well as that of other ternary oxides such as InTaO_4_ and CuWO_4_ (having 2.6 eV^10^ and 2.3 eV^20^ band gap, respectively) requires their modification to narrow their band gap, for an effective solar energy conversion. Attempts to dope these materials with elements such as Ni,^21^ N,^22^ Cr,^22−24^ and Mo^25,26^ proved to increase their visible light absorption capability. In particular, partial substitution of W^6+^ with Mo^6+^ in the CuWO_4_ structure results in a band gap reduction of ca. 0.3 eV (from 2.3 to 2.0 eV),^25^ corresponding to a significant red shift of the absorption onset of the ternary oxide material.^25−27^

Aiming at ascertaining the role that molybdenum for tungsten substitution has in increasing the PEC performance of intrinsically poorly performing CuWO_4_, in relation to the transport properties of the charge carriers photogenerated in differently thick photoactive layers and to the charge transfer efficiency at the oxide-solution interface, in the present work we investigate the CuW_0.5_Mo_0.5_O_4_ semiconductor oxide obtained by 50% Mo^6+^ for W^6+^ substitution, which is quite easy to achieve, the two ions having very similar radii.^28^ This material was prepared by a facile solution-based synthetic route and is composed of a single phase. Its thorough PEC characterization allowed us to demonstrate that the attained reduction in band gap energy directly results in a more efficient exploitation of longer-wavelength photons compared with pure CuWO_4_ photoanodes. The role that molybdenum incorporation has on the efficiencies of photoproduced charge separation in the bulk and charge injection at the electrode/electrolyte interface has been clarified by investigating how the thickness of the photoactive layer in photoanodes affects their wavelength-dependent PEC performance, also in the presence of a suitable hole scavenger.

## 2 Experimental Section

### 2.1 Chemicals and Materials

The following chemicals, all purchased from Sigma-Aldrich, were employed as supplied: copper(II) nitrate trihydrate (99%, Cu(NO_3_)_2_·3H_2_O), ammonium metatungstate hydrate (99%, (NH_4_)_6_H_2_W_12_O_40_·*x*H_2_O), citric acid (99%), boric acid (99%) and ethanol (99%). Molybdenum(VI) oxide bis(2,4-pentanedionate) (99%, C_10_H_14_MoO_6_) was an Alfa Aesar product. Fluorine-doped 2 mm thick tin oxide (FTO) glass was purchased from Pilkington Glass (TEC-7).

### 2.2 Preparation of Photoelectrodes

A 0.5 M solution of CuW_0.5_Mo_0.5_O_4_ was prepared as follows. First, 0.270 g of citric acid, 0.122 g of copper nitrate, 0.062 g of ammonium metatungstate, and 0.082 g of molybdenum oxide bis-pentanedionate were added to 1.0 mL of an ethanol–water 2:1 solution. Complete dissolution of the metal precursors, corresponding to a 1:1 W:Mo molar ratio, was ensured in the chosen solvent composition, and any phase segregation was excluded in the resulting film. The precursors were dissolved by keeping the solution under constant stirring for 45 min at 80 °C. The as-obtained green paste is stable for several weeks. The photoelectrodes were prepared by spin coating it onto a 2.5 × 2.5 cm^2^ FTO glass at 4000 rpm for 30 s. Prior to deposition, the FTO glass was cleaned by 30 min-long sonication in a soap solution, followed by careful washing, sonication in ethanol for 30 min, and drying in air. The clean glass slices then underwent a 15 min-long UV-cleaner ozone treatment to remove any organic species deposited onto the FTO surface. They were finally soaked in isopropanol for a few seconds right before the spin coating deposition, to increase the FTO affinity for the metal oxide precursor solution and reduce the light scattering of the resulting films in the long-wavelength spectral region.

After deposition, the CuW_0.5_Mo_0.5_O_4_ films were dried at 250 °C for 10 min, followed by annealing at 550 °C for 1 h. Pure CuWO_4_ films were prepared by a similar procedure without adding the molybdenum precursor to the initial solution. Multilayer films (up to 5 layers), labeled as *n*L (*n* = 1–5) CuW_0.5_Mo_0.5_O_4_, were prepared by coating the as-deposited films with the same precursor solution, followed by annealing at 550 °C for 1 h after each deposition step.

### 2.3 Optical, Structural, Morphological, and Photoelectrochemical Tests

UV–visible absorption spectra were recorded in the transmission mode using a Jasco V-670 spectrophotometer. The crystalline phase of the materials was investigated through X-ray powder diffraction (XRPD) analysis using a Philips PW1820 diffractometer, equipped with a Cu-sealed tube that provided Kα radiation at 40 mA and 40 kV. A model LEO 1430 scanning electron microscope operating at a 10 kV accelerating voltage and at 8 mm working distance was used to acquire the top view and cross-sectional images of the films, up to three deposited layers. A Dektak XT Bruker profilometer was employed to measure the thickness of the thicker 4L and 5L CuW_0.5_Mo_0.5_O_4_ films.

Linear sweep voltammetry (LSV) measurements were carried out using a three-electrode cell equipped with two quartz windows. The FTO/CuW_0.5_Mo_0.5_O_4_ film was used as working electrode, an Ag/AgCl (3.0 M NaCl) as reference electrode, and a platinum gauze as counter electrode. The electrical bias was swept at 10 mV s^–1^ using an Autolab PGSTAT 12, controlled by the NOVA software. The light source was an Oriel, Model 81172 solar simulator equipped with an AM 1.5 G filter. The light intensity, measured by means of a Thorlabs PM200 power meter equipped with a S130VC power head with Si detector, was 100 mW cm^–2^. During a typical LSV test, 5 consecutive *J–V* scans were performed with each electrode, preceded by the Fermi level equilibrium under irradiation up to the open circuit potential (OCP). Identical OCP values were recorded prior to the beginning of each scan with each mono- or multilayer electrode and all of them provided stable and reproducible photocurrent from the first to the last scan.

The investigated films were tested as photoanodes under both back (through the FTO side) and front (through the deposited film side) irradiation configuration, in contact with a 0.1 M K_3_BO_3_ aqueous solution at pH 9.^29^ The buffer borate solution was prepared by adding KOH to aqueous boric acid up to the desired pH. Furthermore, a series of different sacrificial agents, acting as electron donor species, was employed in LSV measurements. Aqueous solutions (0.1 M) of either H_2_O_2_, NH_3_, or NaNO_2_ were buffered at pH 9 in a K_3_BO_3_ solution, while the 0.1 M Na_2_SO_3_ aqueous solution did not contain K_3_BO_3_ as it is naturally at pH 9. The potential values versus Ag/AgCl were converted into the RHE scale using the following equation: *E*_RHE_ = *E*_AgCl_ + 0.059 pH + *E*°_AgCl_, with *E*°_AgCl_ (3.0 M NaCl) = 0.210 V at 25 °C.

Incident photon to current efficiency (IPCE) measurements were carried out at 1.23, 1.5, and 1.7 V versus RHE under irradiation with a 300 W Lot-Oriel Xe lamp equipped with a Lot-Oriel Omni-λ 150 monochromator and a Thorlabs SC10 automatic shutter, in the above-described single-compartment three-electrode cell containing the K_3_BO_3_ buffered solution, to which the NaNO_2_ hole scavenger was eventually added. The IPCE values were calculated using the following equation:
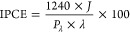
1where *J* is the photocurrent density (mA cm^–2^) and *P*_λ_ (mW cm^–2^) is the power measured at each specific wavelength λ (nm).

Chopped chronoamperometric scans at different wavelengths were recorded in back side configuration at 1.23 V versus RHE within the 300–650 nm wavelength range, with a 10 nm step. A 420 nm filter was employed at λ > 500 nm, to avoid any contribution from the high-order harmonics originated from the monochromator.

## 3 Results and Discussion

### 3.1 Characterization of Photoelectrodes

**Figure 1:**
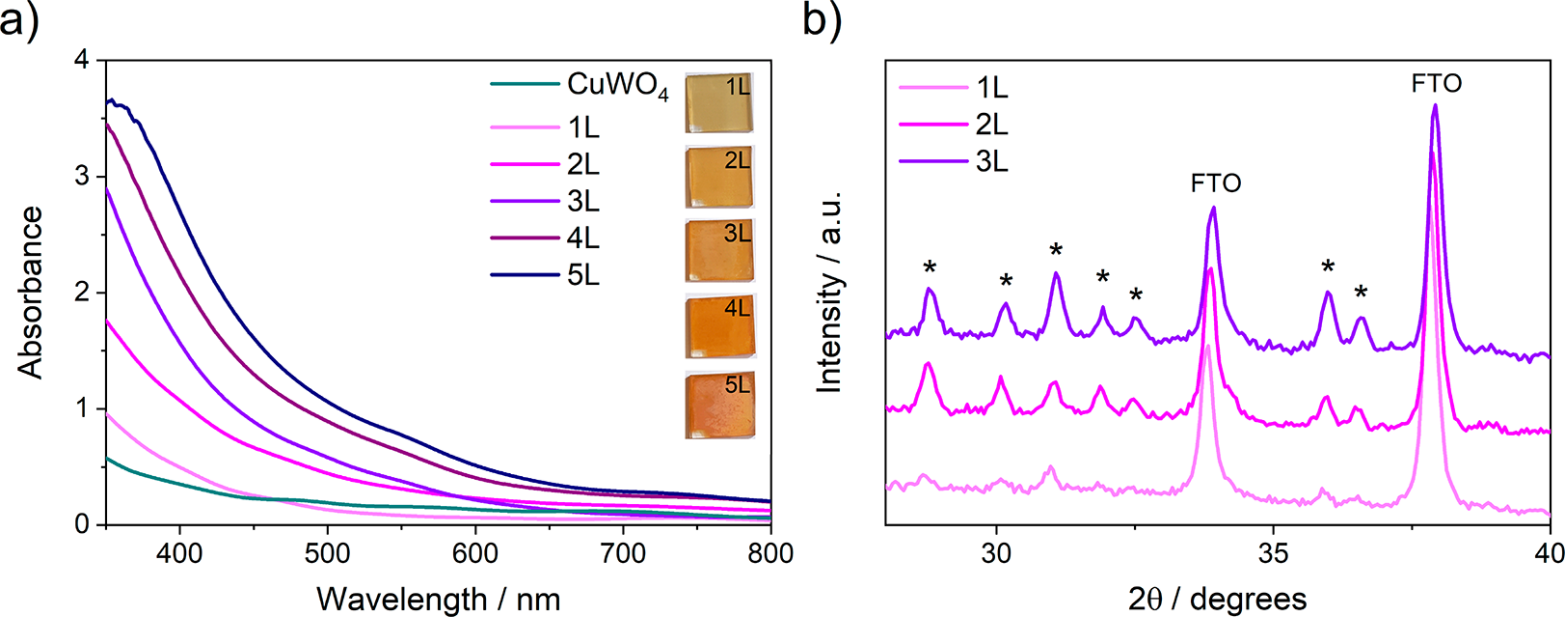
(a) Absorption spectra of multilayer (1L–5L) CuW_0.5_Mo_0.5_O_4_ photoelectrodes. Inset: picture of the photoelectrodes. (b) X-ray powder diffraction (XRPD) patterns of 1L, 2L, and 3L CuW_0.5_Mo_0.5_O_4_ electrodes, in the 28–40 degrees 2θ range. The asterisks mark the reflections typical of wolframite.

As shown in Figure 1a, the multilayer CuW_0.5_Mo_0.5_O_4_ electrodes exhibit an absorption increasing with the increasing number of deposited layers, with an absorption tail due to light scattering observed with the thickest films. The gradual color increase can be appreciated also from their pictures shown in the inset of Figure 1a. The absorption onset of the material is at ca. 650 nm, corresponding to a band gap of ca. 1.9 eV, in agreement with the ca. 2.0 eV estimated band gap reported for a 50% degree of Mo for W substitution.^25^ Thus, the absorption onset of pure copper tungstate, having a band gap energy of 2.3 eV,^30^ is effectively extended toward the visible light region upon 50% Mo for W substitution.

The XRPD patterns relative to the 1L, 2L, and 3L CuW_0.5_Mo_0.5_O_4_ films, reported in Figure 1b, show the reflections characteristic of pure wolframite,^25,26^ which become more intense with increasing material thickness. Thus, 50% Mo for W substitution has no effects on the crystalline phase formation, within the detection limits of the XRPD technique. The presence of Mo is confirmed by the change of lattice constants and the consequent peak shifts observed in the XRPD patterns of CuW_0.5_Mo_0.5_O_4_ in comparison with CuWO_4_ (see a detailed view of Figure 1b reported in Figure S1 of the Supporting Information), which are almost identical to those recently reported^26^ for a CuW_1–*x*_Mo_*x*_O_4_ sample with the same nominal Mo:W molar ratio.

**Figure 2:**
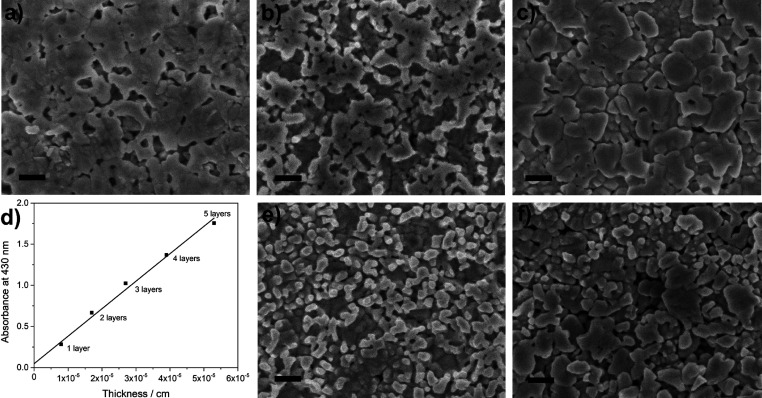
Top-view SEM images of (a) 1L, (b) 2L, and (c) 3L CuW_0.5_Mo_0.5_O_4_ electrodes. (d) Absorption at 430 nm of multilayer photoanodes vs their thickness. SEM images recorded after PEC tests of (e) 2L and (f) 3L CuW_0.5_Mo_0.5_O_4_ electrodes. The scalebar is 200 nm.

The top view scanning electron microscopy (SEM) images of 1L, 2L, and 3L CuW_0.5_Mo_0.5_O_4_ electrodes (Figure 2a–c) reveal a structure composed of densely aggregated crystallites, which may facilitate the electrolyte diffusion across the film by increasing the contact area between the electrolyte solution and the photoactive material. In the case of the thickest film, a decrease in the size of the agglomerates can be observed in the top view SEM images acquired with the same electrode after PEC tests (see Figure 2e,f), resulting from an extended contact between the CuW_0.5_Mo_0.5_O_4_ film and the electrolyte.

The thickness values of the multilayer CuW_0.5_Mo_0.5_O_4_ electrodes, evaluated either by means of SEM cross-sectional images (Figure S2) or by profilometry, are reported in Table 1 and indicate that the average increase in film thickness is ca. 110 nm upon each deposited layer. From these values, together with the absorption spectrum of the films, the absorption coefficients of CuW_0.5_Mo_0.5_O_4_ at different wavelengths were calculated according to the Lambert–Beer law, as the slopes of the lines obtained by plotting the absorbance of the film at a selected wavelength against the film thickness of multilayer CuW_0.5_Mo_0.5_O_4_ electrodes (see Figures 2d and S3). The best fitting was obtained for λ = 430 nm, the corresponding absorption coefficient being α_430 nm_ = (3.41 ± 0.10) × 10^4^ cm^–1^.

**Table 1:** Thickness of Multilayer CuW_0.5_Mo_0.5_O_4_ Electrodes Estimated from SEM Cross-Sectional Images and by Profilometry

sample	thickness/nm
1L	80 ± 20
2L	170 ± 10
3L	270 ± 10
4L	410 ± 20
5L	530 ± 30

### 3.2 PEC Performances

**Figure 3:**
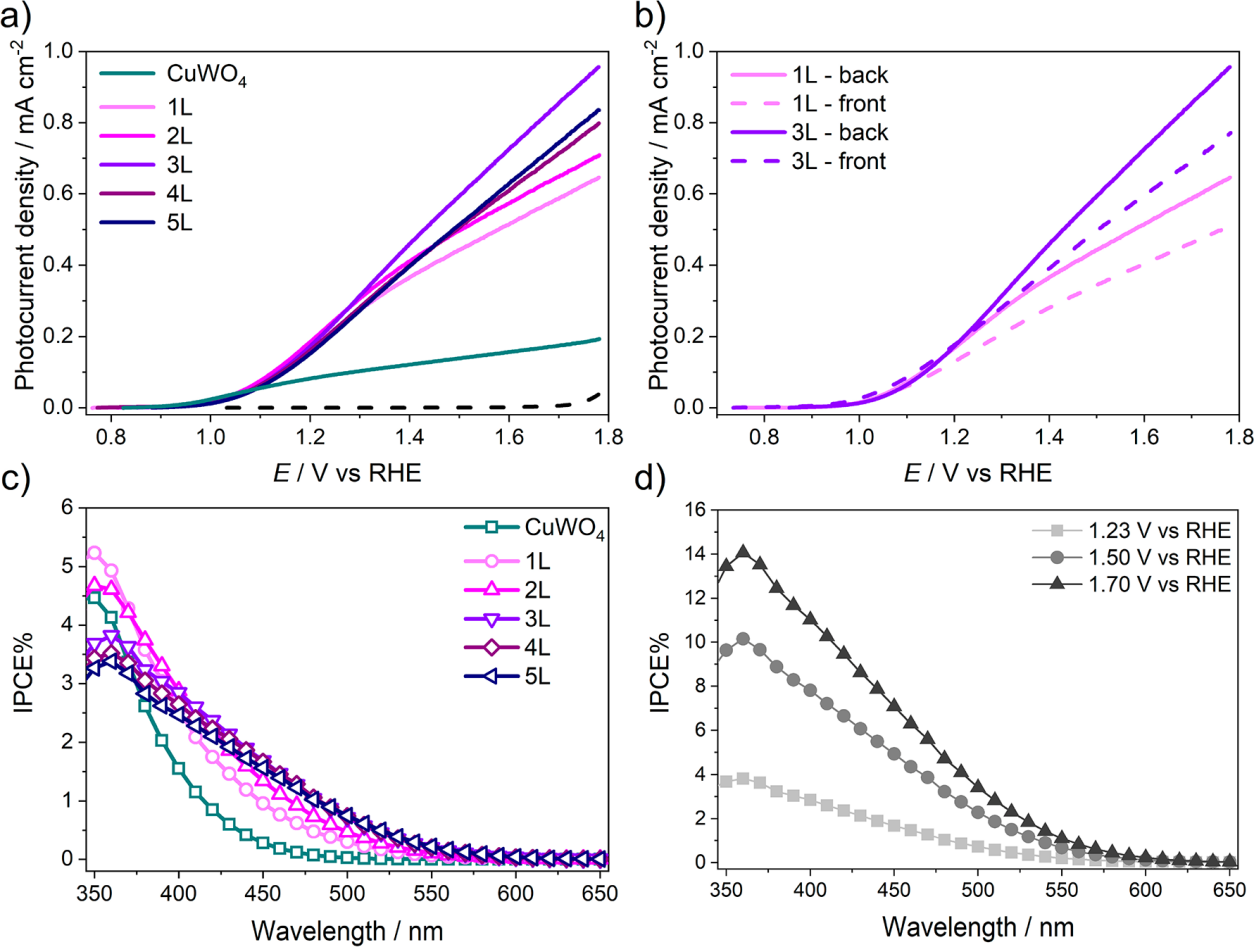
Linear sweep voltammetry (LSV) of (a) 1L–5L CuW_0.5_Mo_0.5_O_4_ electrodes and CuWO_4_ electrode in back configuration and (b) 1L and 3L CuW_0.5_Mo_0.5_O_4_ electrodes in back and front configuration; AM 1.5 G solar simulated irradiation, scan rate of 10 mV s^–1^. The current in the absence of irradiation is also shown in panel (a) (black dashed line). Incident photon to current efficiency (IPCE) of (c) CuWO_4_ monolayer and 1L–5L CuW_0.5_Mo_0.5_O_4_ electrodes at 1.23 V vs RHE and (d) the 3L electrode at different applied potentials.

During a typical LSV test under simulated solar light irradiation, five consecutive photocurrent density versus applied potential (*J*–*V*) scans were performed with each electrode. The *J*–*V* curves reported in Figure 3a,b correspond to the fifth scan performed with each electrode. Each mono- or multilayer electrode provided stable and reproducible photocurrent from the first to the last scan, with no difference in photocurrent, as shown in Figure S4. Thus, high repeatability was obtained with all films, in line with the high photostability of CuWO_4_-based materials.

Figure 3a shows the *J*–*V* plots recorded with the multilayer CuW_0.5_Mo_0.5_O_4_ electrodes and a monolayer CuWO_4_ electrode under back side solar simulated light (i.e., the electrodes were irradiated through the FTO glass), in contact with a 0.1 M K_3_BO_3_ solution at pH 9. First of all, the introduction of Mo into CuWO_4_ produces a more than 3-fold improvement in photocurrent density at 1.7 V versus RHE. Moreover, no significant difference in the LSV curves of CuW_0.5_Mo_0.5_O_4_ films could be noted up to an applied potential of ca. 1.3 V versus RHE, while progressively higher photocurrent density values were recorded at higher applied potentials with increasing number of deposited layers up to the 3L CuW_0.5_Mo_0.5_O_4_ electrode, which was found to be the most active one.

Consequently, the highest photocurrent density of ca. 1 mA cm^–2^ was recorded at 1.7 V versus RHE for the 3L film, while lower performances were obtained with thicker 4L and 5L electrodes, despite that they practically absorb 100% of the incident light, also at long wavelengths. This provides a first indication of electron mobility issues occurring in films composed of more than three layers (i.e., thicker than ca. 270 nm). Furthermore, the slightly higher activity resulting for the 5L film compared with the 4L film could be related to the contribution to photocurrent of the longest-wavelength photons, the exploitation of which increases with increasing film thickness, that is, with increasing absorption of long-wavelength radiation (see Figure S5, showing a magnification of the IPCE profile of Figure 3c).

The *J*–*V* curves of Figure 3a were compared with the results of LSV analyses performed under front-side illumination (Figure S6; i.e., by irradiating the material through the electrode/electrolyte interface). This allows us to get more insight into the charge-carrier mobility within the material and to assess if the internal charge transport is limited by either the minority or the majority charge carriers,^31^ that is, by the holes or the electrons, respectively, in an n-type semiconductor material.^32^ The LSV plots recorded with the 1L and 3L CuW_0.5_Mo_0.5_O_4_ electrodes (ca. 80 and 270 nm thick, respectively) under back and front side irradiation are compared in Figure 3b. Since for both selected films the photocurrent recorded under front side irradiation is lower than that recorded under back side irradiation, poor electron transport appears to be the limiting factor for this material. The 3L electrode exhibits the best PEC performance among the tested films either under front or back irradiation (compare Figure 3a and Figure S6). Thus, a ca. 270 nm film thickness seems to correspond to the best balance between photon absorption in the photoactive layer and electron transport efficiency within it.

The IPCE plots recorded under back side irradiation at 1.23 V versus RHE are reported in Figure 3c for the complete series of multilayer CuW_0.5_Mo_0.5_O_4_ electrodes and for a single-layer CuWO_4_ photoanode. By comparing the curves obtained with the two monolayer CuWO_4_ and CuW_0.5_Mo_0.5_O_4_ photoanodes, evidence is obtained of the higher efficiency of the composite molybdenum-containing material with respect to pure CuWO_4_, over the entire range of investigated wavelengths. This demonstrates that the increased visible light absorption capability (see Figure 1a) consequent to Mo for W substitution results in a higher number of photogenerated charge couples, which are effectively responsible for the higher visible light activity of CuW_0.5_Mo_0.5_O_4_ with respect to CuWO_4_.

Furthermore, by comparing the IPCE curves obtained with multilayer CuW_0.5_Mo_0.5_O_4_ electrodes (Figure 3c), the thinnest 1L and 2L CuW_0.5_Mo_0.5_O_4_ films are most efficient in the UV region up to 390 nm, whereas thicker films exhibit the best photoactivity in the visible light region. The high absorption coefficient value of CuW_0.5_Mo_0.5_O_4_ in the UV region (the extinction coefficient at 400 nm is 3 times greater than at 500 nm, see Figure S7) implies that UV radiation is absorbed almost quantitatively even by the thinner films, which results in high photocurrent values. Furthermore, in thicker electrodes (thicker than 2L) and under back side irradiation, most of the UV photogenerated holes need to travel across the whole film to reach their extraction sites at the film/electrolyte interface. The probability that they recombine with photopromoted electrons increases with increasing film thickness leading to lower IPCE values in the UV region with respect to thinner films. Concerning the relatively higher IPCE values of the thicker films in the visible region, owing to the lower absorption capability of the material at longer wavelengths (Figure S7), quantitative light harvesting can be attained only in thicker films, which are consequently able to better exploit visible light. The best compromise between efficient hole transport across the film and maximum visible light exploitation under back side irradiation is attained with the ca. 270 nm thick film (3L CuW_0.5_Mo_0.5_O_4_ electrode).

The recorded IPCE values are also in good agreement with the performances under full lamp irradiation recorded in LSV scans. In fact, as shown in Table S1, there is a good matching between the photocurrent density values at 1.23 V versus RHE in *J*–*V* curves and the photocurrent density calculated by integrating the product between the IPCE curves and the standard AM 1.5 G solar spectrum^33^ over the entire range of investigated wavelengths (300–650 nm for the CuW_0.5_Mo_0.5_O_4_ multilayer films).

Besides at 1.23 V versus RHE, the IPCE curve for the best performing 3L CuW_0.5_Mo_0.5_O_4_ electrode was recorded in back side configuration also at 1.50 and 1.70 V versus RHE (Figure 3d), in order to evaluate the conversion efficiency of the material upon increasing charge separation. As shown in Figure 3d, the conversion efficiency gets higher with increasing applied potential over the whole investigated wavelengths, with the IPCE value recorded at 400 nm showing a 2.5- and 3.5-fold enhancement as the potential increases from 1.23 to 1.5 and to 1.7 V versus RHE, respectively. The observed behavior is in line with the photocurrent density enhancement recorded in LSV scans under simulated solar light irradiation. In particular, the IPCE almost linearly increases with increasing applied potential (see Figure 3d), because the holes generated by high-energy photons, which are mainly confined in the proximity of the FTO back contact, benefit from the enhanced charge carrier separation because of the progressively higher external bias and have larger probability to reach the film/electrolyte interface where O_2_ evolution occurs.

**Figure 4:**
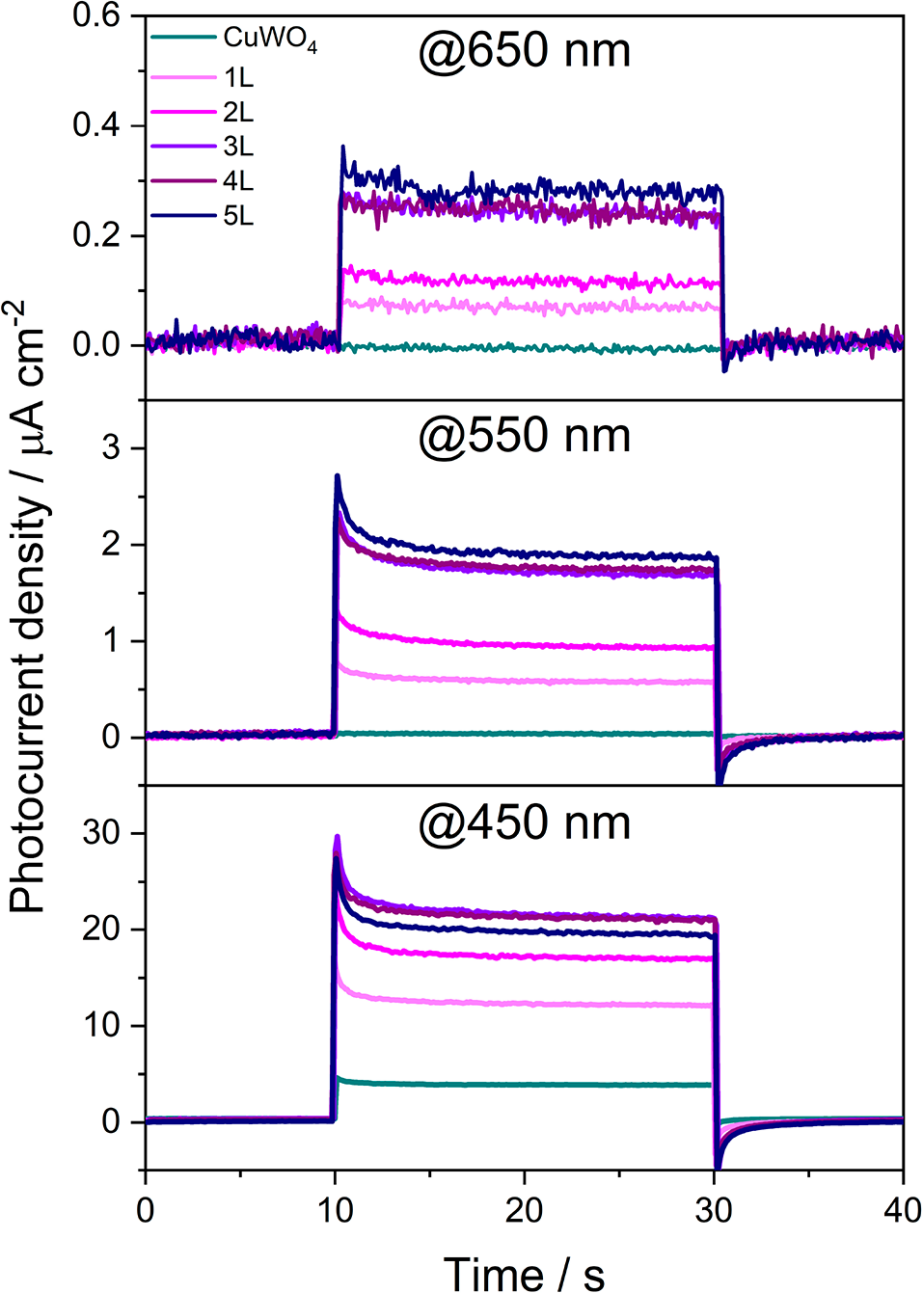
Chopped chronoamperometry at 1.23 V versus RHE of the 1L–5L CuW_0.5_Mo_0.5_O_4_ electrodes and of the CuWO_4_ electrode under irradiation at 650 nm (top panel), 550 nm (middle panel), and 450 nm (bottom panel).

Finally, the chopped photocurrent measurements under monochromatic irradiation at different wavelengths performed with our photoanodes at 1.23 V versus RHE provide uncontroversial evidence that all CuW_0.5_Mo_0.5_O_4_-based photoanodes absorb light and exhibit photoactivity up to 650 nm, while CuWO_4_ is photoactive only below 550 nm. In fact, as shown in Figure 4, whereas all tested photoanodes, including the CuWO_4_-based one, originate a photocurrent signal under irradiation at 450 nm (Figure 4, bottom panel), the photocurrent signal is zero for CuWO_4_ under irradiation at 550 nm (Figure 4, middle panel), while all 1L–5L CuW_0.5_Mo_0.5_O_4_ photoanodes remain photoactive at this wavelength and maintain their photoactivity up to 650 nm (Figure 4, top panel). This demonstrates that Mo for W substitution in CuWO_4_ largely extends the photoactivity of this material into the visible region, at least up to 650 nm.

### 3.3 Sacrificial Agents

Two are the main limiting factors for PEC water oxidation performed by semiconductor-based photoanodes, that is, (i) the transport of photopromoted electrons and photogenerated holes through the bulk material to the FTO back contact and to the film/electrolyte interface, respectively, and (ii) the interfacial hole injection kinetics, determining the oxygen evolution efficiency. Decoupling these two contributions is therefore required in order to shed light on the intrinsic properties of the investigated photoactive material. In particular, the use of a suitable hole scavenger-containing electrolyte, acting as electron donor, allows one to study the intrinsic properties of the bulk material, under the assumption that hole injection at the material/electrolyte interface is not rate limiting under such conditions.^34^

Previous studies pointed out that significant dark current is observed if CuWO_4_ electrodes are in contact with Na_2_SO_3_- and H_2_O_2_-containing solutions.^35^ Therefore, the performance of CuW_0.5_Mo_0.5_O_4_ photoanodes was investigated in the presence of various hole scavengers (i.e., Na_2_SO_3_, H_2_O_2_, NaNO_2_, and NH_3_) to check the existence of dark currents and verify if and how the response of this material is affected by the redox potential of the donor species. The concentration of all sacrificial agents was kept constant at 0.1 M within 0.1 M K_3_BO_3_ buffer solutions at pH 9, except for the 0.1 M Na_2_SO_3_ solution, which was at pH 9 in the absence of buffer.

The dark current onsets in the presence of different sacrificial agents was measured first using both the 1L CuW_0.5_Mo_0.5_O_4_ film and a glassy carbon as working electrode, in order to attain an apparent redox scale for the employed species (see Figure S8). For the electrode immersed in 0.1 M K_3_BO_3_ in the absence of any hole scavenger, the dark current potential onset is the highest (i.e., around 1.8 V versus RHE). Furthermore, the most easily oxidizable species is H_2_O_2_, immediately followed by Na_2_SO_3_, with a dark current onset located at ca. 1.0 V versus RHE. More positive onset potentials (i.e., ca. 1.4 and 1.6 V versus RHE) were found for NaNO_2_ and NH_3_, respectively. Thus, moving from the most stable to the most oxidizable species, the following scale results: H_2_O (i.e., K_3_BO_3_ aqueous solution) > NH_3_ > NaNO_2_ ≫ Na_2_SO_3_ > H_2_O_2_.

**Figure 5:**
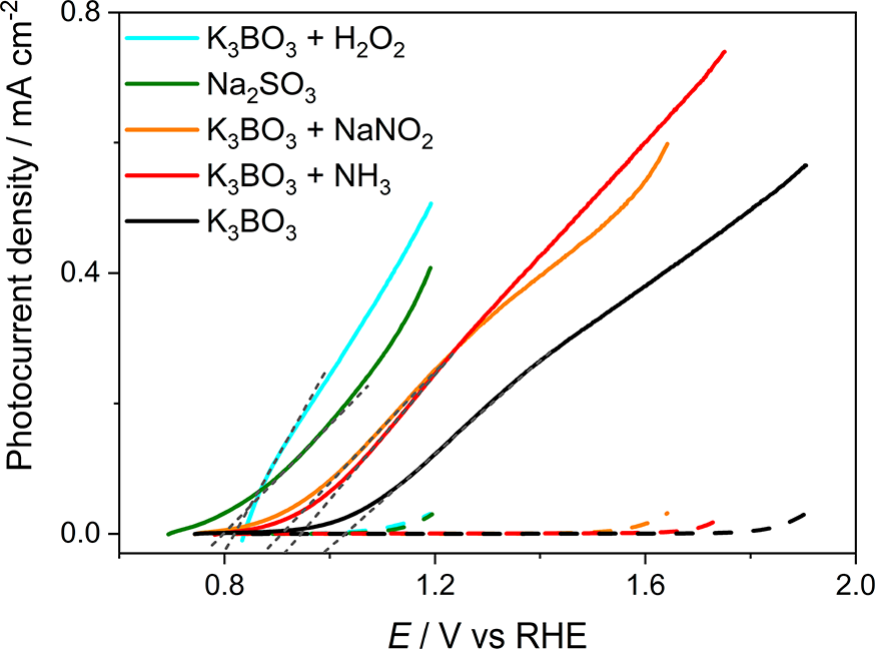
LSV curves recorded with a monolayer CuW_0.5_Mo_0.5_O_4_ electrode in contact with a 0.1 M K_3_BO_3_ solution, 0.1 M K_3_BO_3_ solutions containing different hole scavengers at a 0.1 M concentration or 0.1 M Na_2_SO_3_, under dark (dashed lines) or back side AM 1.5 G irradiation conditions (continuous lines). Scan rate of 10 mV s^–1^. All solutions were at pH 9. The photocurrent onset potential was calculated by extrapolation of each LSV line (black dashed lines).

The LSV curves obtained with a monolayer CuW_0.5_Mo_0.5_O_4_ electrode in contact with the hole scavenger-containing solutions under back side simulated solar irradiation are shown in Figure 5. The LSV curve obtained with the 0.1 M K_3_BO_3_ aqueous solution is also reported, as reference. In the absence of any electron donor species, the photocurrent onset potential was ca. 1.0 V versus RHE (i.e., 800 mV lower than under dark conditions). The photocurrent onset potentials in the presence of the hole scavengers, extrapolated from the curves shown in Figure 5, are reported in Table S2. Compared with the pure K_3_BO_3_ electrolyte solution, the use of Na_2_SO_3_ and H_2_O_2_ as hole scavengers led to the largest decrease of photocurrent onset potential, which was around 0.8 V versus RHE in both cases, in good agreement with the conduction band edge value of ca. 0.7 V versus RHE.^25^ However, owing to the significant dark current generated in the presence of these sacrificial agents at a potential as low as 1.2 V versus RHE, Na_2_SO_3_ and H_2_O_2_ were discarded as hole scavengers in our PEC investigation.

Additionally, in the presence of NH_3_ and NaNO_2_ hole scavengers, a lower negative shift of the photocurrent onset potential (located at ca. 0.9 V versus RHE) was observed and negligible dark current up to 1.75 and 1.6 V versus RHE, respectively. This allows the exploitation of a wider applied potential window in PEC measurements. Because of the high volatility of NH_3_, which may result in uncontrollable concentration variation of its aqueous solutions, we finally selected NaNO_2_ as the most suitable sacrificial agent for our PEC tests. In its presence, the photocurrent density at 1.23 V versus RHE is double compared with that measured in pure K_3_BO_3_ (0.30 versus 0.15 mA cm^–2^, see Figure 5), with negligible dark current at such applied bias.

Anyway, regardless of the employed electron donor, the photocurrent just moderately increased with respect to water oxidation (in K_3_BO_3_). This is in contrast with the considerable increase typically observed with semiconductors the PEC performance of which is limited by surface hole accumulation and slow water oxidation kinetics. For instance, BiVO_4_ photoanodes generate up to 100 times higher current in contact with a hole-scavenger-containing solution,^36^ whereas Fe_2_O_3_ electrodes in contact with H_2_O_2_ allow the complete consumption of surface holes.^37^ This suggests that the transfer of surface holes to water or electron donor species has minor limiting effects on the PEC efficiency of CuW_0.5_Mo_0.5_O_4_.

**Figure 6:**
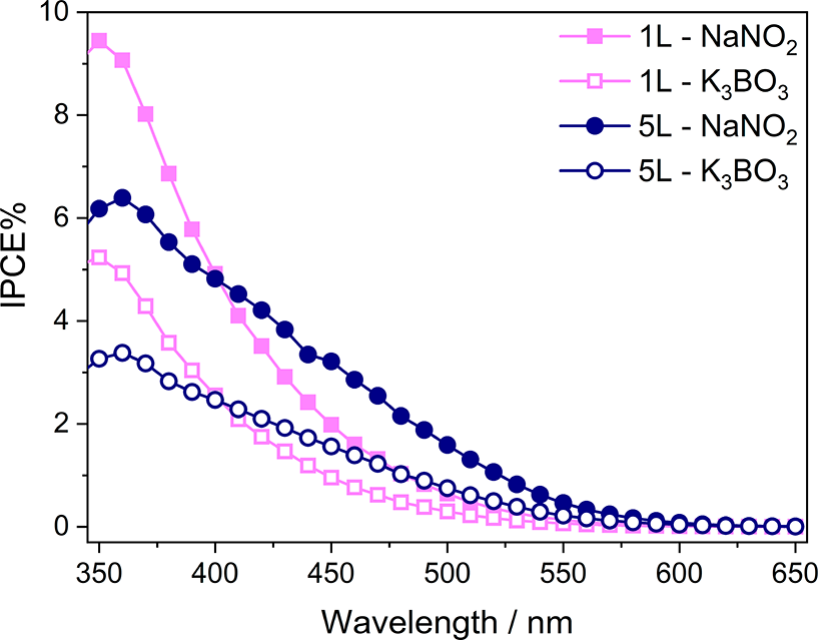
Incident photon to current efficiency (IPCE) of 1L and 5L CuW_0.5_Mo_0.5_O_4_ electrodes at 1.23 V vs RHE in K_3_BO_3_ both in the absence (void symbols) and in the presence (full symbols) of NaNO_2_.

The IPCE curves obtained at 1.23 V versus RHE with the 1L and 5L CuW_0.5_Mo_0.5_O_4_ electrodes in the presence of the NaNO_2_ hole scavenger (Figure 6) evidence the extended visible light photoactivity of the CuW_0.5_Mo_0.5_O_4_ material, which is maximum for the thickest 5L photoanode.

### 3.4 Charge Separation and Charge Injection Efficiencies

PEC measurements in the presence of NaNO_2_ as hole scavenger allowed us to evaluate the charge separation and charge injection efficiencies and thus assess if the performance of CuW_0.5_Mo_0.5_O_4_ photoanodes is limited by either the material bulk properties or the surface hole injection kinetics.

The photocurrent density *J* can be expressed as the combination of different contributions, according to eq 2:^37,38^
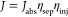
2where *J*_abs_ is the theoretical maximum photocurrent density obtained if all absorbed photons are converted into electricity, which was estimated by converting into current the integral of the product between the standard AM 1.5 G solar spectrum and the absorption spectrum of the photoelectrode over the 300–650 nm range (4.39 mA cm^–2^ for our 1L CuW_0.5_Mo_0.5_O_4_ material),^15^ η_sep_ is the charge separation efficiency, and η_inj_ is the interfacial charge injection efficiency. From this equation the charge separation and charge injection efficiencies can be easily calculated by taking into account the photocurrent densities recorded in the presence (*J*_NaNO_2__) and absence (*J*_K_3_BO_3__) of the hole scavenger, as follows:
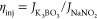
3
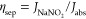
4

**Figure 7:**
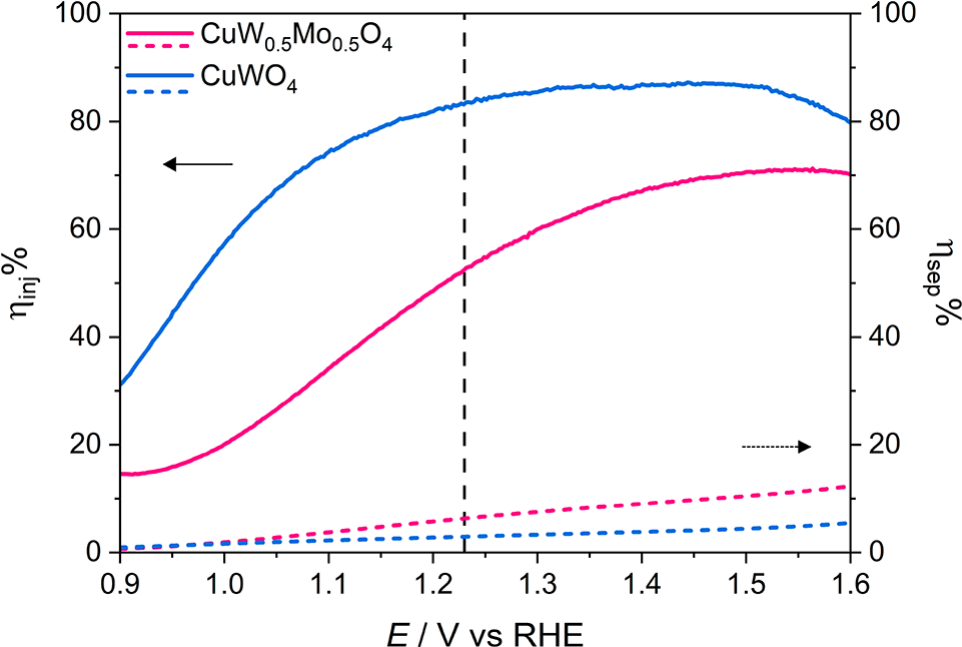
Charge injection efficiency η_inj_ (continuous lines) and charge separation efficiency η_sep_ (dashed lines) calculated for the monolayer CuW_0.5_Mo_0.5_O_4_ (fuchsia) and CuWO_4_ (light blue) electrodes vs the applied potential.

The calculated η_inj_ and η_sep_ values for the 1L CuW_0.5_Mo_0.5_O_4_ electrode and for the CuWO_4_ electrode are shown in Figure 7 as a function of the applied potential. At 1.23 V versus RHE η_inj_ and η_sep_ are 52% and 6%, respectively, for 1L CuW_0.5_Mo_0.5_O_4_, which clearly indicate that charge separation in the bulk material is the main limiting factor for the PEC performance of our investigated material, in line with the limited photoactivity improvement attained in the presence of electron donor species.

For our CuWO_4_ photoanode (with *J*_abs_ amounting to 3.60 mA cm^–2^ in the 300–550 nm range) η_inj_ = 83% and a η_sep_ = 3% at 1.23 V versus RHE. This implies that Mo incorporation into CuWO_4_ induces a 2-fold increase of the charge separation properties in the bulk, in line with recent reports,^27^ while it negatively affects the hole injection efficiency at the CuW_0.5_Mo_0.5_O_4_ film/electrolyte interface, with a η_inj_ decrease.

The increased charge separation efficiency can result from the increased majority carrier (i.e., electron) concentration in Mo-doped CuWO_4_ with respect to CuWO_4_ evidenced through Mott–Schottky plots in previous studies on Mo-doped CuWO_4_.^25−27^ This would improve the material conductivity and thus reduce charge recombination in CuW_0.5_Mo_0.5_O_4_ films. Additionally, the reduction in charge injection efficiency may be ascribed to a Mo-induced increase of the surface trap states of CuWO_4_,^12,29,35,39,40^ which would decrease the efficiency of water oxidation especially at relatively low applied potentials, by inducing Fermi level pinning at the semiconductor–liquid junction. An indirect evidence of the increase of surface trap states in CuW_0.5_Mo_0.5_O_4_ films is the modest delay in photocurrent onset of the LSV curves recorded with CuW_0.5_Mo_0.5_O_4_-based photoanodes with respect to the CuWO_4_ photoanode appearing in Figure 3a and better evidenced in the enlargement of this figure at low potentials shown in Figure S9. As recently outlined,^27^ such delay is compatible with an increase in surface trap states upon Mo incorporation, which hampers the photocurrent onset at low overpotentials, that is, when the driving force to separate photoproduced electron–hole couples is small. Taken together, the 2-fold improvement in charge separation and the decrease in charge injection efficiency result in the overall enhanced PEC activity of CuW_0.5_Mo_0.5_O_4_ with respect to CuWO_4_.

## 4 Conclusions

Substitution of Mo for W into the CuWO_4_ structure is responsible for the conduction band edge energy lowering and consequent band gap narrowing. Thus, the increased PEC performance of CuW_0.5_Mo_0.5_O_4_ with respect to CuWO_4_ mainly results from the extended visible light absorption ability and photoactivity of the CuW_0.5_Mo_0.5_O_4_ material up to 650 nm. This effect is maximum for the highest film thickness explored in this study ensuring the exploitation of long-wavelength photons in the visible region, which are efficiently harvested for longer optical paths within the photoactive films. However, Mo for W substitution also increases the efficiency of photoproduced charge separation, which is due to an increased conductivity of the material, while the lower charge injection efficiency of CuW_0.5_Mo_0.5_O_4_ with respect to pure CuWO_4_ may result from an increased amount of surface trap states. With its 2.0 eV band gap, copper molybdo-tungstate shows promise as photoanode material for practical water splitting. Further improvements are needed and might be attained through nanostructuring, the development of efficient oxygen evolution cocatalysts for this class of materials, and the fine modification of the electronic structure by doping with other elements.

## Supporting Information Available

Details of XRPD spectra, cross-section SEM images, absorption coefficient and band gap (Tauc plot) determination, magnification of IPCE data, LSV under front side irradiation, dark current onset with different electrolytes, experimental and calculated (from IPCE integration) photocurrents (PDF)
