# Supplementary material for: Effective Visible Light Exploitation by Copper Molybdo-tungstate Photoanodes
Source: ACS Appl Energy Mater. 2020 Jun 8;3(7):6956–64. doi: 10.1021/acsaem.0c01021 (PMC8016397; doi:10.1021/acsaem.0c01021)
Supplement: Supplementary file 1 — ae0c01021_si_001.pdf [file ae0c01021_si_001.pdf]

Supporting Information

Effective Visible Light Exploitation by Copper Molybdo-tungstate Photoanodes

Annalisa Polo, Chiara Nomellini, Ivan Grigioni, Maria Vittoria Dozzi, and Elena Selli*

Dipartimento di Chimica, Università degli Studi di Milano, via Golgi 19, I-20133 Milano, Italy

E-mail: elena.selli@unimi.it

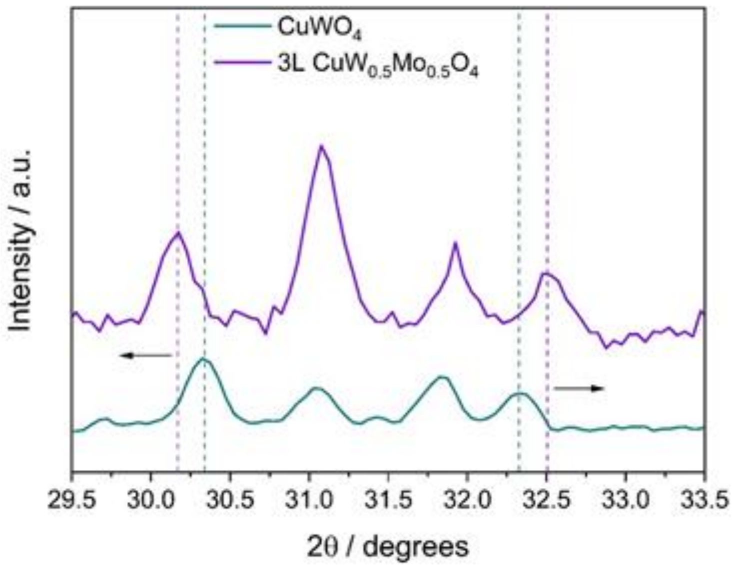

Figure S1. Detailed view of the XRPD pattern in the 29.5° – 33.5° 2θ range of $CuWO_4$ (green) and 3L $CuW_{0.5}Mo_{0.5}O_4$ (violet), to be compared with Figure 1b of ref. 26 of the main text.

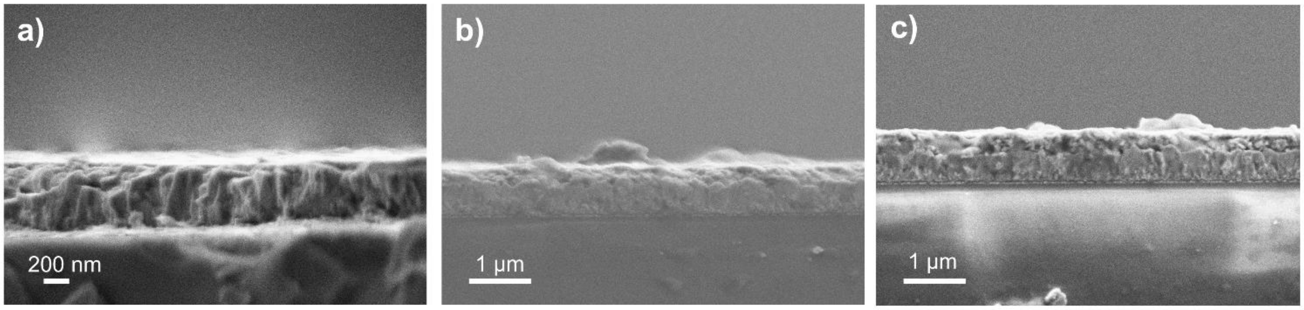

Figure S2. Cross-section SEM images of (a) 1L, (b) 2L and (c) 3L $CuW_{0.5}Mo_{0.5}O_4$ electrodes.

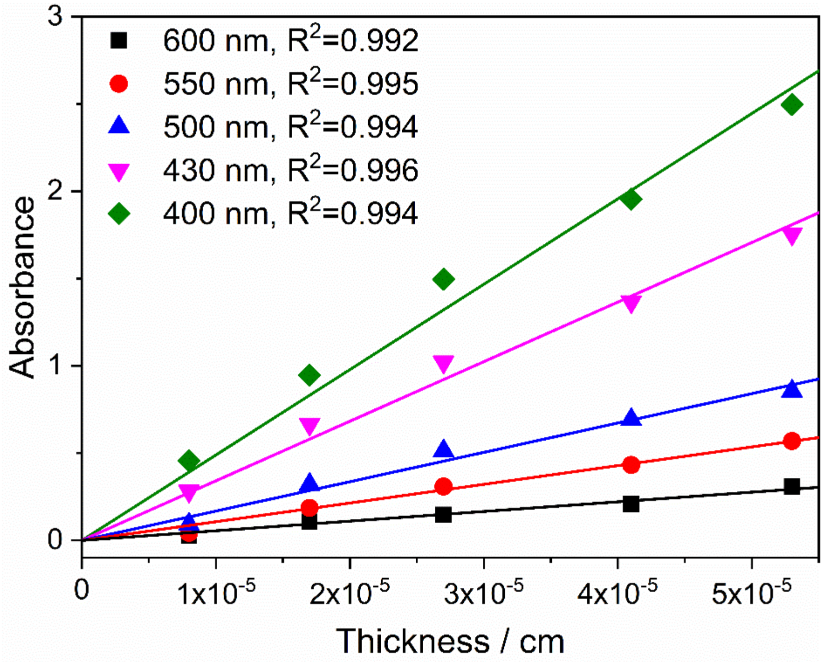

Figure S3. Absorbance of the multilayer (1L–5L) $CuW_{0.5}Mo_{0.5}O_4$ photoanodes at various wavelengths *vs.* their thickness, and R^2 values for each regression line. The calculated absorption coefficients are $\alpha_{400nm} = (4.89 \pm 0.16)\cdot10^4$ cm^{-1}, $\alpha_{430nm} = (3.41 \pm 0.10)\cdot10^4$ cm^{-1}, $\alpha_{500nm} = (1.68 \pm 0.06)\cdot10^4$ cm^{-1}, $\alpha_{550nm} = (1.07 \pm 0.03)\cdot10^4$ cm^{-1}, $\alpha_{600nm} = (0.55 \pm 0.02)\cdot10^4$ cm^{-1}.

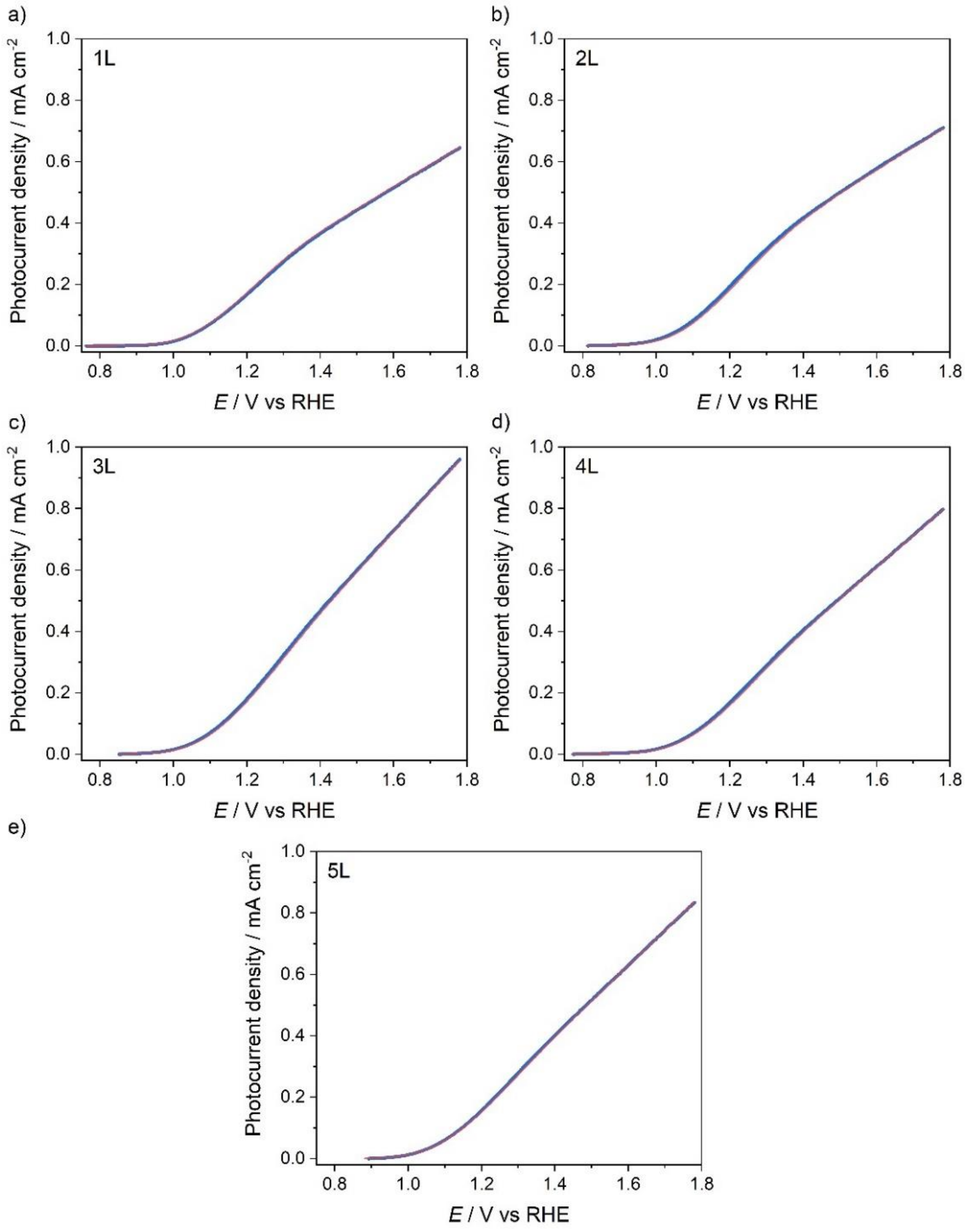

Figure S4. First and fifth LSV scan under simulated solar light irradiation recorded with (1L–5L) $CuW_{0.5}Mo_{0.5}O_4$ photoanodes.

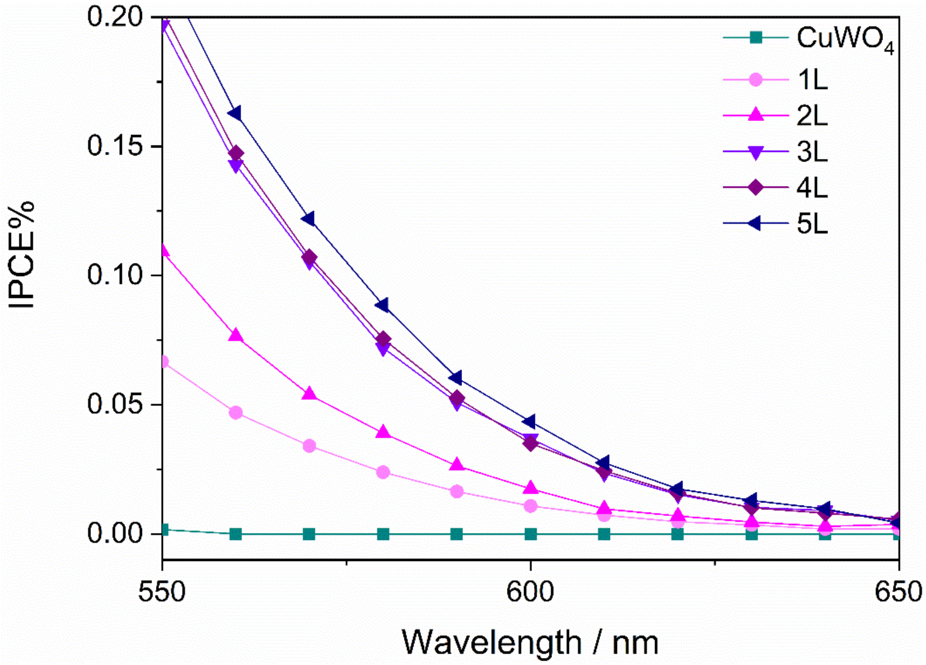

Figure S5. Magnification at the longest wavelengths region of the IPCE profiles of $CuWO_4$ monolayer and 1L–5L $CuW_{0.5}Mo_{0.5}O_4$ electrodes at 1.23 V *vs.* RHE.

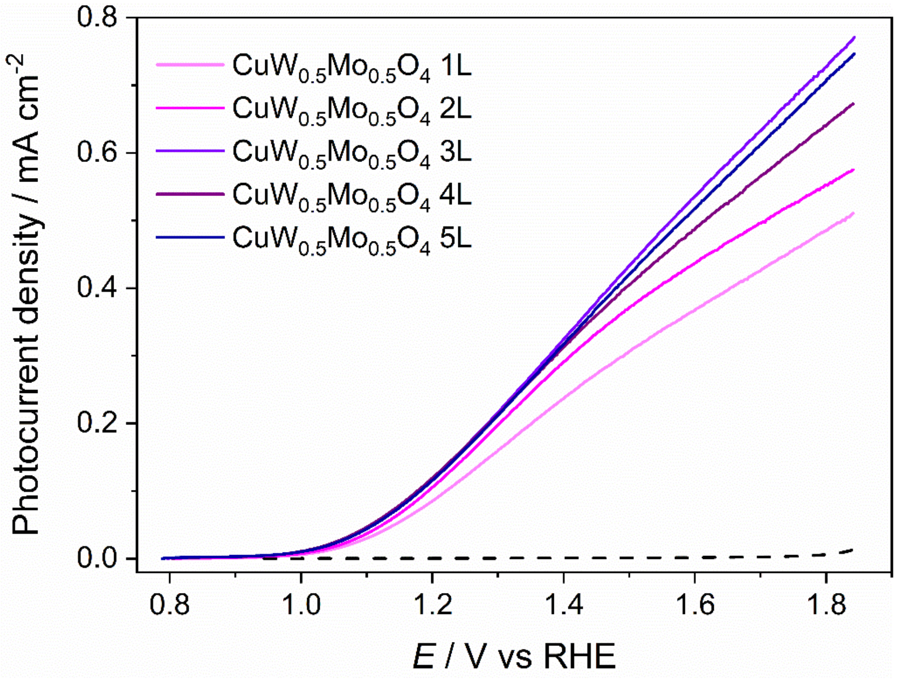

Figure S6. Linear Sweep Voltammetry (LSV) recorded with the 1L–5L $CuW_{0.5}Mo_{0.5}O_4$ electrodes under front side AM 1.5 G irradiation. Scan rate 10 mV s^{-1}.

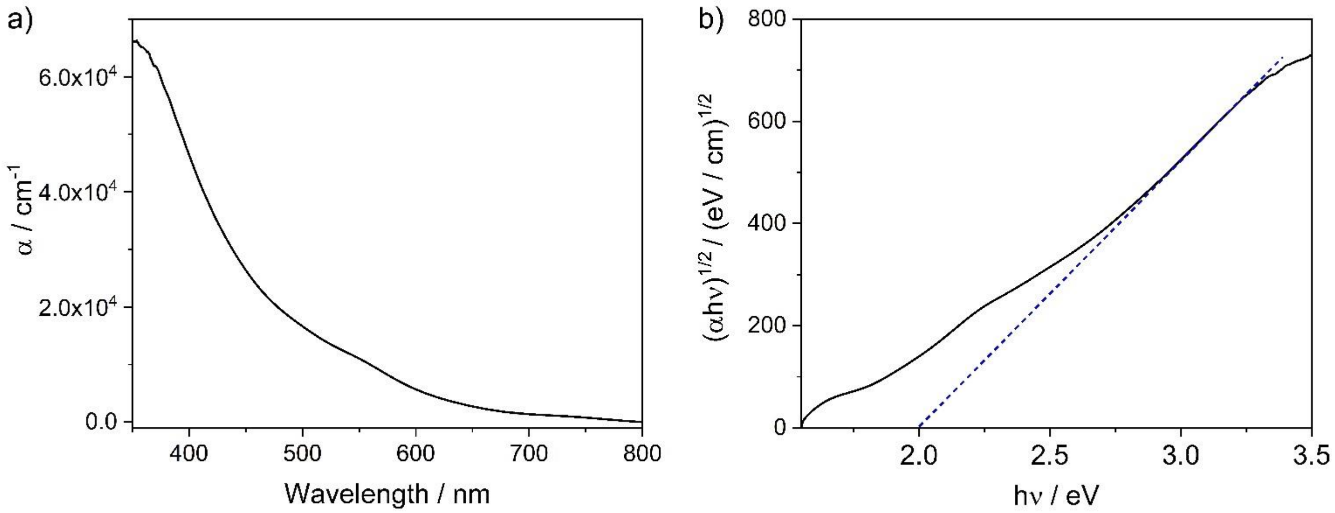

Figure S7. (a) Absorption coefficient of $CuW_{0.5}Mo_{0.5}O_4$ in the 300–800 nm wavelength range; (b) corresponding Tauc plot analysis.

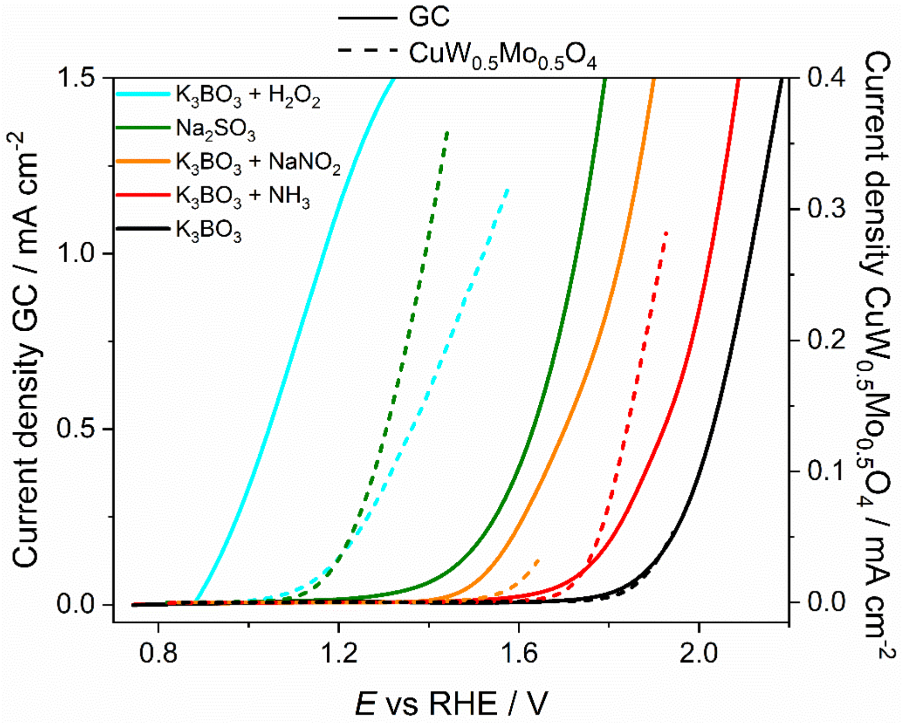

Figure S8. Dark current recorded in the presence of the employed sacrificial agents using a glassy carbon (GC, continuous lines) or our 1L $CuW_{0.5}Mo_{0.5}O_4$ sample (dashed lines) as working electrode.

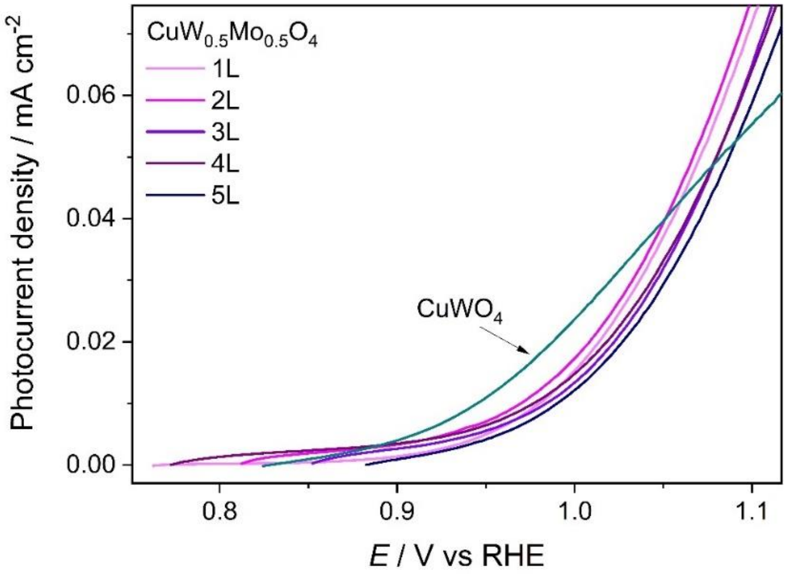

Figure S9. Magnification of Figure 3a at low potentials, evidencing the delay in photocurrent onset of the LSV curves recorded with $CuW_{0.5}Mo_{0.5}O_4$-based photoanodes with respect to the LSV curve of the $CuWO_4$ photoanode.

Table S1. Photocurrent density measured at 1.23 V *vs.* RHE and integrated photocurrent values calculated from IPCE analyses at the same applied potential for $CuW_{0.5}Mo_{0.5}O_4$ multilayer electrodes.

Sample	Measured photocurrent / mA cm^{-2}	Integrated photocurrent / mA cm^{-2}
1L	0.107	0.104
2L	0.132	0.119
3L	0.145	0.127
4L	0.147	0.123
5L	0.143	0.118

Table S2. Photocurrent onset potential values extrapolated from LSV plots recorded in different solutions (see Figure 5 of the main text).

**Electrolyte solution**	**Photocurrent onset potential / eV**
K_3BO_3	1.03
NH_3	0.95
$NaNO_2$	0.91
H_2O_2	0.82
Na_2SO_3	0.79
